# Social Work Graduates Entering the Field of Gerontology During COVID-19: Exploring Perceived Readiness and Needs

**DOI:** 10.1093/geroni/igab046.1511

**Published:** 2021-12-17

**Authors:** Susanny Beltran, Vivian Miller

**Affiliations:** 1 University of Central Florida, Orlando, Florida, United States; 2 Bowling Green State University, Bowling Green, Ohio, United States

## Abstract

Gerontological social workers (GSW) are on the frontlines supporting the biopsychosocial needs of older adults in hospitals, communities, and long-term care settings. However, it is unclear whether social workers (SW) are trained to meet the emerging needs of older adults during COVID-19. This study describes training received, perceived readiness, and training needs of GSWs new to the field during 2020. A cross-sectional online survey was conducted with recent graduates from U.S. SW programs. Survey questions explored training received and ongoing needs, perceived self-efficacy (adapted from the Geriatric Social Work Competency Scale), demographics, and confidence in ability to work with populations 55+. A total of 15 recent SW graduates specializing in gerontology completed the survey. Fifty-three percent of the sample held MSWs and over half (53.3%) were licensed social workers or registered interns. Nearly all participants (73.3%) reported taking an introductory aging course, and almost half (46.7%) completed coursework in aging and diversity, aging policy, and end-of-life care/bereavement; 80% completed fieldwork in aging. Participants report moderate skill in assessing issues related to losses or transitions (46.7%), and physical functioning (53.3%), and advanced skill in assessing cognitive functioning (60.0%), and caregiver stress/needs (53.3%). Nearly half of respondents who rated their training as good-excellent indicate being very-extremely confident (42.8%) in their ability to practice with older adults. Training needs among participants include disaster preparedness, telehealth, and coordination of scarce resources. Curriculum development and continuing education are necessary to support emerging gerontological social workers in their practice during COVID-19 and other emergencies.

